# Amelanotic nodular melanoma in Marjolin ulcer on the plantar region^☆^

**DOI:** 10.1016/j.abd.2021.08.015

**Published:** 2023-03-13

**Authors:** Valentina Lourenço Lacerda de Oliveira, Lucia Martins Diniz, Karla Spelta, Elton Almeida Lucas

**Affiliations:** Hospital Universitário Cassiano Antônio Moraes, Universidade Federal do Espírito Santo, Vitória, ES, Brazil

Dear Editor,

This case describes an 80-year-old female Caucasian patient, who sought dermatological evaluation for a lesion on the left foot after local trauma, with progressive growth over two years. Dermatological examination showed a nodular, vegetating, tumor-like lesion covered by hematic crusts on the left plantar region, overlapping an extensive area of deformities in the foot anatomy, caused by healing by secondary intention of burn lesions that occurred in childhood (Fig. 1). Histopathology showed an ulcer filled with a fusiform cell proliferation arranged in bundles in different directions, occupying the superficial and deep dermis, and moderate cell pleomorphism (Fig. 2). Immunohistochemistry was positive for S-100 protein, gp100 and Melan-A, confirming the diagnosis of melanoma (Fig. 3). The association of clinical and laboratory findings led to the diagnosis of acral amelanotic nodular melanoma on a burn scar. The authors found in the literature four case reports of amelanotic melanoma arising on chronic scars when searching Pubmed using the terms “burn and amelanotic melanoma” and “Marjolin ulcer and amelanotic melanoma”, demonstrating the rarity of such a presentation.

**Figure 1:**
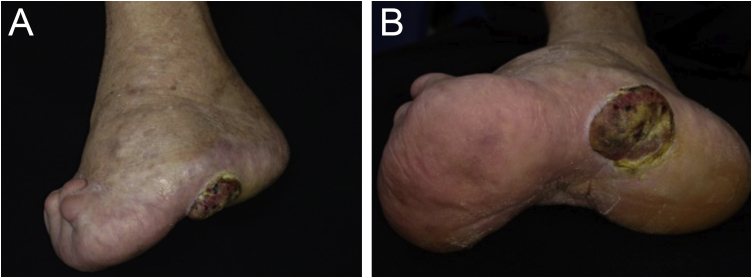
(a) Anatomical deformity in the left foot and absence of toes, secondary to a burning accident in childhood, that healed by second intention. (b) Vegetating nodular-tumor lesion with hematic crusts, well-defined borders, blackened spots, and overlying keratosis, affecting the plantar region and lateral face of the left foot.

**Figure 2:**
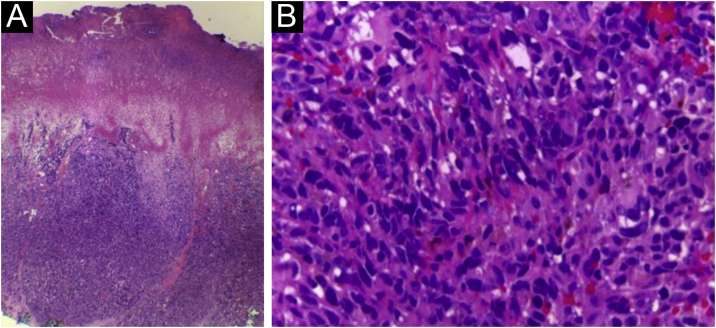
(a) Ulcerated lesion with cell proliferation occupying the superficial and deep dermis. (b) Moderately pleomorphic, fusiform and epithelioid cells, with hyperchromatic nuclei; nucleoli are difficult to distinguish.

**Figure 3:**
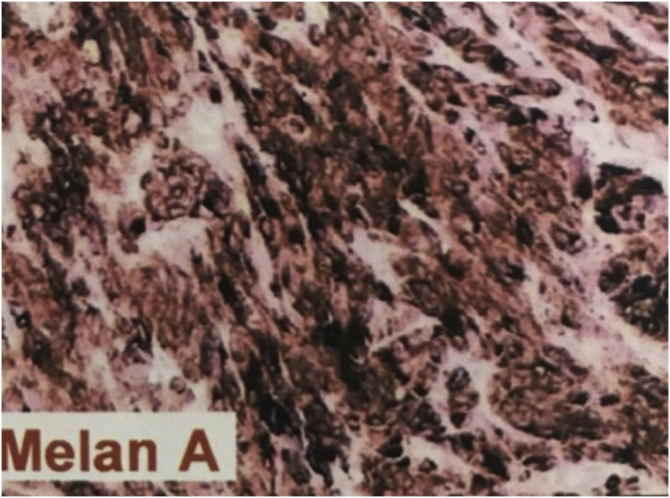
Immunohistochemistry was positive for Melan-A monoclonal antibody, which recognizes a specific melanocytic differentiation protein expressed in benign and malignant melanocytes.

The lesion was completely excised, as well as the ipsilateral lymph nodes, after confirmation of a positive sentinel lymph node for metastatic melanoma. Imaging exams showed alterations suggestive of metastasis in face and skull bones. The patient died after eight months.

Marjolin ulcer is a rare and aggressive tumor, occurs mainly in the fifth decade of life, and is prevalent in the male sex.^1^ It arises over scar tissue, particularly after burns, occurring in 0.7% to 2% of these lesions.^1^, ^2^, ^3^ Clinically, the lesions arise over previous scarring, as a non-healing, ulcerated or hardened, rapidly growing lesion that may become exophytic and bleeding. The most frequent histopathological type of neoplasia arising in Marjolin ulcers is squamous cell carcinoma (80%–90%), followed by basal cell carcinoma (9.6%) and, rarely, melanoma (2.4%).^1^, ^2^, ^3^ The low incidence of melanoma in these scars can be explained by the small number of melanocytes in the scar tissue.^1^

In the epidemiology of melanomas, the nodular type represents the second most frequent one (approximately 15% of diagnosed melanomas), and 5% of nodular melanomas are amelanotic.^4^

Amelanotic acral melanoma has an incidence of 1.8% of melanoma cases,^4^ and sometimes manifests as hyperkeratotic lesions.^2^, ^5^

The diagnoses of Marjolin ulcer and melanoma are defined by clinical history, physical examination, and histopathology. The therapeutic options depend on TNM staging, including excision surgery with wide margins or limb amputation, in addition to chemotherapy and radiotherapy.^1^

Scars are susceptible to trauma due to a lack of collagen organization and vascular supply compromised by fibrosis, which obstructs vessels. Virchow's theory attributes the development of malignancy in scars to repeated trauma in the area, which leads to chronic irritation, repeated re-epithelialization, and local damage to the skin immune system, with a reduction in Langerhans cells and production of toxins in the affected area, in addition to genetic predisposition.^1^, ^2^, ^3^

A rare manifestation of Marjolin ulcer is presented in this case, which presented as a nodular acral amelanotic melanoma, reinforcing the need for advice to patients, care, and regular follow-up of burn scars, for early diagnosis and treatment, reducing morbidity and mortality.

## Financial support

None declared.

## Authors' contributions

Valentina Lourenço Lacerda de Oliveira: Design and planning of the study; drafting and editing of the manuscript; collection, analysis, and interpretation of data; critical review of the literature; critical review of the manuscript.

Lucia Martins Diniz: Approval of the final version of the manuscript; effective participation in research orientation; intellectual participation in the propaedeutic and/or therapeutic conduct of the studied cases; critical review of the manuscript.

Karla Spelta: Intellectual participation in the propaedeutic and/or therapeutic conduct of the studied cases; critical review of the manuscript.

Elton Almeida Lucas: Intellectual participation in the propaedeutic and/or therapeutic conduct of the studied cases; critical review of the manuscript.

## Conflicts of interest

None declared.
